# Cuprizone Affects Hypothermia-Induced Neuroprotection and Enhanced Neuroblast Differentiation in the Gerbil Hippocampus after Ischemia

**DOI:** 10.3390/cells9061438

**Published:** 2020-06-10

**Authors:** Woosuk Kim, Kyu Ri Hahn, Hyo Young Jung, Hyun Jung Kwon, Sung Min Nam, Tae Hyeong Kim, Jong Whi Kim, Dae Young Yoo, Dae Won Kim, Jung Hoon Choi, Yeo Sung Yoon, In Koo Hwang

**Affiliations:** 1 Department of Anatomy and Cell Biology, College of Veterinary Medicine, and Research Institute for Veterinary Science, Seoul National University, Seoul 08826, Korea; tank3430@naver.com (W.K.); hkinging@snu.ac.kr (K.R.H.); hyoyoung@snu.ac.kr (H.Y.J.); kjwfirst@gmail.com (J.W.K.); 2 Department of Biomedical Science and Research Institute for Bioscience and Biotechnology, Hallym University, Chuncheon 24252, Korea; 3 Department of Biochemistry and Molecular Biology, Research Institute of Oral Sciences, College of Dentistry, Gangneung-Wonju National University, Gangneung 25457, Korea; donuts25@gwnu.ac.kr (H.J.K.); kimdw@gwnu.ac.kr (D.W.K.); 4 Department of Anatomy, College of Veterinary Medicine, Konkuk University, Seoul 05030, Korea; lovingvet@gmail.com; 5 Department of Anatomy, College of Veterinary Medicine and Institute of Veterinary Science, Kangwon National University, Chuncheon 24341, Korea; xogudsla9402@kangwon.ac.kr (T.H.K.); jhchoi@kangwon.ac.kr (J.H.C.); 6 Department of Anatomy, College of Medicine, Soonchunhyang University, Cheonan 31151, Korea; dyyoo@sch.ac.kr

**Keywords:** cuprizone, ischemia, hippocampus, neurogenesis, hypothermia

## Abstract

In the present study, we investigated the effects of cuprizone on cell death, glial activation, and neuronal plasticity induced by hypothermia after ischemia in gerbils. Food was supplemented with cuprizone at 0.2% ad libitum for eight weeks. At six weeks after diet feeing, gerbils received transient forebrain ischemia with or without hypothermic preconditioning. Cuprizone treatment for 8 weeks increased the number of astrocytes, microglia, and pro-inflammatory cytokine levels in the hippocampus. In addition, cuprizone treatment significantly decreased the number of proliferating cells and neuroblasts in the dentate gyrus. Brain ischemia caused cell death, disruption of myelin basic proteins, and reactive gliosis in CA1. In addition, ischemia significantly increased pro-inflammatory cytokines and the number of proliferating cells and differentiating neuroblasts in the dentate gyrus. In contrast, hypothermic conditioning attenuated these changes in CA1 and the dentate gyrus. However, cuprizone treatment decreased cell survival induced by hypothermic preconditioning after ischemia and increased the number of reactive microglia and astrocytes in CA1 as well as that of macrophages in the subcallosal zone. These changes occurred because the protective effect of hypothermia in ischemic damage was disrupted by cuprizone administration. Furthermore, cuprizone decreased ischemia-induced proliferating cells and neuroblasts in the dentate gyrus.

## 1. Introduction

Cerebral ischemia is a major cause of disability, acute mortality, chronic morbidity, and death worldwide [1]. This condition results from the reduction or loss of oxygen supply, and prolonged ischemia may lead to insidious delayed degeneration of specific vulnerable neurons within the affected brain region, such as the hippocampus [2,3,4]. Oxidative stress plays a key role in the pathophysiological cascade, leading to brain tissue injury. In particular, ischemia followed by reperfusion causes a rapid, transient increase in the production of reactive oxygen species (ROS) [5]. The resulting accumulation of free radicals within the tissue can result in DNA damage and lipid peroxidation [6,7,8]. Mitochondrial damage activates intracellular apoptotic signaling pathways and ultimately induces the activation of cysteine proteases of the caspase family [9,10]. Antioxidants have neuroprotective effects against ischemic damage because they neutralize ROS production and mitochondrial dysfunction [11,12,13,14].

Hypothermic therapy prevents neuronal damage from ischemia through various mechanisms [15,16,17,18]. In the early phase, hypothermia affects the alteration of cerebral blood flow, preservation of energy stores, and reduction of excitatory amino acids. In the later phase, it prevents apoptotic death via the inhibition of inflammation and reduction in blood–brain barrier disruption [19]. In addition, mild hypothermia decreases neuronal damage by reducing the production of ROS and preserving the respiratory chain in mitochondria [20]. Hypothermia also increases the survival of newly generated cells in the granule cell layer of the dentate gyrus after ischemia [21,22]. However, in aged rats, hypothermia does not enhance neurogenesis, although the blood vessels are increased in the peri-infarct area [23].

Copper, a cofactor of various cuproenzymes, plays crucial roles in several cellular processes. Disturbances of copper levels in the nervous system result in severe neuronal degeneration [24]. In particular, the cuproenzyme cuprizone (bis-cyclohexanone-oxalyldihydrazone) causes demyelination in the hippocampus [25,26] and decreases the activity of cytochrome oxidase and other mitochondrial enzymes such as monoamine oxidase in the brain [27]. In addition, cuprizone impairs the respiratory chain (complex IV) and increases the formation of ROS in neurons [28,29]. Increased ROS levels facilitate inflammatory responses and ultimately cause neuronal death. Treatment with cuprizone for six weeks was shown to significantly increase cerebral infarction after middle cerebral artery occlusion in mice compared to that in a control group [30]. Administration of cuprizone for five weeks induced complete demyelination in the brain with reactive gliosis and axonal damage [31]. In a previous study, we observed that cuprizone treatment significantly decreased the expression of myelin basic protein (MBP) in the corpus callosum and the number of proliferating cells and differentiating neuroblasts in the dentate gyrus [25]. However, only a few studies examining the effects of demyelination on hypothermia-induced neuroprotection against ischemic damage have been reported.

In the present study, therefore, we investigated the effects of hypothermia on cuprizone-induced demyelination and/or cell death and subsequent activation of astrocytes and microglia in the hippocampal CA1 region. We also investigated the effects of hypothermia on cell proliferation and neuroblast differentiation in the dentate gyrus of gerbils after demyelination induced by cuprizone, which significantly decreases hippocampal neurogenesis in the dentate gyrus, as described in a previous study [25].

## 2. Materials and Methods

### 2.1. Experimental Animals

Six-week-old male Mongolian gerbils were purchased from Japan SLC, Inc. (Shizuoka, Japan). Gerbils were housed at an adequate temperature (22 ± 2 °C) and humidity (60 ± 5%), with a 12-h light/12-h dark cycle and provided ad libitum access to food and tap water. The handling and care of animals conformed to current international laws and policies (NIH Guide for the Care and Use of Laboratory Animals, NIH Publication No. 85-23, 1985, revised in 1996) and was approved by the Institutional Animal Care and Use Committee of Kangwon National University (KW-170727-3). All experiments were conducted with an effort to minimize both the number of animals used and the physiological stress caused by the procedures employed.

### 2.2. Experimental Groups and Treatments

**Figure 1:**
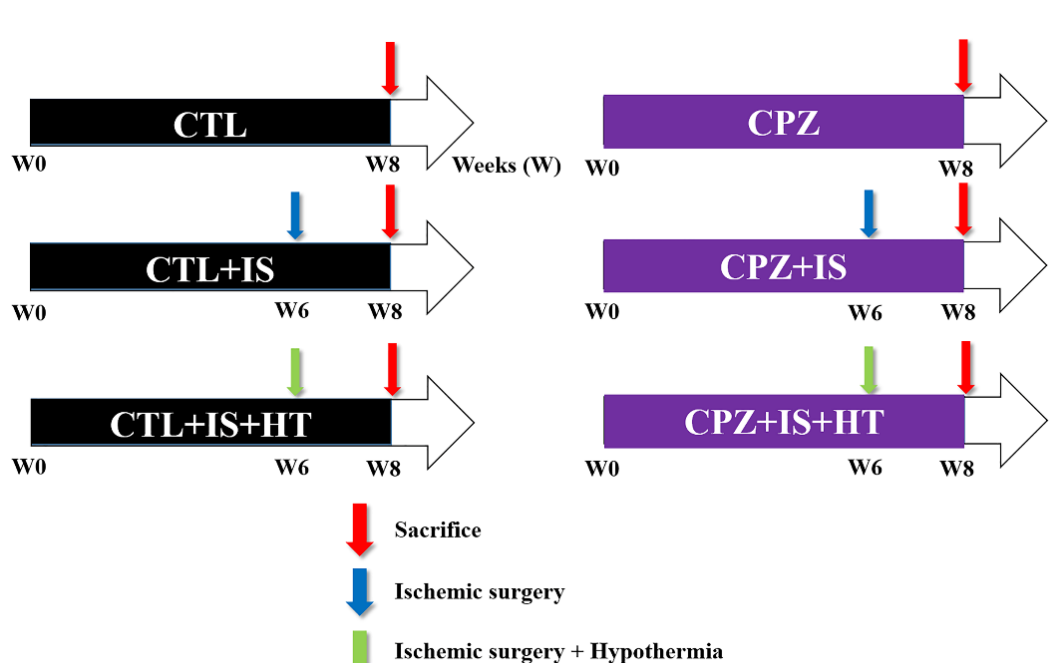
Experimental design of the treatment protocol in normal diet (CTL), cuprizone diet (CPZ), CTL + normothermic ischemic brain surgery (IS), CPZ + IS, CTL + IS + hypothermic treatment (HT), and CPZ + IS + HT groups.

Gerbils (*n* = 10 in each group) were randomly divided into six groups: groups 1 and 2 did not undergo ischemic brain surgery and had a normal diet (CTL group) and a cuprizone diet for eight weeks (CPZ group), respectively. Groups 3 and 4 underwent normothermic ischemic brain surgery after six weeks of a normal diet (CTL + IS group) and a cuprizone diet (CPZ + IS group), respectively. Groups 5 and 6 underwent hypothermic ischemic brain surgery after six weeks of a normal diet (CTL + IS + HT group) and a cuprizone diet (CPZ + IS + HT group), respectively, as shown in Figure 1. Cuprizone diets were prepared by adding 0.2% cuprizone to chow diets, and animals continued their respective diets for two weeks after surgery, at which time they were sacrificed.

### 2.3. Induction of Transient Forebrain Ischemia

The animals were anesthetized with a mixture of 2.5% isoflurane (Baxter, Deerfield, IL, USA) in 33% oxygen and 67% nitrous oxide. Common carotid arteries were isolated and occluded bilaterally using non-traumatic aneurysm clips as previously described [32]. Complete interruption of blood flow was confirmed by observing the central artery in the retinae using an ophthalmoscope (HEINE K180^®^; HEINE Optotechnik, Herrsching, Germany). Aneurysm clips were removed after 5 min of occlusion. In groups 3 and 4, body temperature in each animal in the ischemic surgery groups was monitored under free-regulating or normothermic (37 ± 0.5 °C) conditions with a rectal temperature probe (TR-100; Fine Science Tools, Foster City, CA, USA) and maintained using a thermometric blanket pre-, intra-, and postoperatively, until the animals completely recovered from anesthesia. In groups 5 and 6, body temperature was maintained under hypothermic (33.5 ± 0.5 °C) conditions. One animal was excluded due to incomplete occlusion of the common carotid arteries. Groups 1 and 2 underwent the same surgical procedure without the occlusion of common carotid arteries (sham operation).

### 2.4. Tissue Processing

The animals were anesthetized with a mixture of alfaxalone (Alfaxan, 75 mg/kg; Careside, Seongnam, Korea) and xylazine (10 mg/kg; Bayer Korea, Seoul, Korea) two weeks after ischemia/reperfusion. Subsequently, the thoracic cavity was opened and perfused transcardially with 0.1 M phosphate buffered saline (PBS, pH 7.4) followed by 4% paraformaldehyde in 0.1 M phosphate buffer (PB, pH 7.4) using a flexible tube (HV-06409-16, Masterflex, Vernon Hills, IL, USA) with a needle as described previously [25,32]. The brains were then dissected and post-fixed for 12 h in the same fixative. Brain tissue was cryoprotected by overnight infiltration with 30% sucrose in 0.1 M PB. Tissue sections from the region between 1.4 and 2.0 mm caudal to the bregma (with reference to a gerbil atlas [33]) were selected. Serial coronal sections (30 μM in thickness) were made using a cryostat (Leica, Wetzlar, Germany) and collected in six-well plates containing PBS for further processing. Sections were processed under the same conditions to ensure that histological data were comparable among the groups.

### 2.5. Cresyl Violet Staining

Sections were stained with cresyl violet acetate to detect surviving neurons in the hippocampus. Three sections from the region 1.4 to 2.0 mm caudal to the bregma (with reference to a gerbil atlas [33]), separated by intervals of 180 μM, were mounted on gelatin-coated microscope slides. Cresyl violet acetate (Sigma, St. Louis, MO, USA) was dissolved in distilled water at 1% with glacial acetate at 0.25% and used to immerse the slides for 5 min. Slides were washed in distilled water for 5 min before and after staining. Subsequently, sections were dehydrated with ethanol, cleared with xylene (3 × 10 min), and mounted using Canada balsam (Kanto Chemical, Tokyo, Japan).

### 2.6. Immunohistochemistry for Glial Fibrillary Acidic Protein (GFAP), Ionized Calcium-Binding Adapter Molecule 1 (Iba-1), MBP, Mac3, Ki67, and Doublecortin (DCX)

Three sections from the region 1.4 to 2.0 mm caudal to the bregma (with reference to a gerbil atlas [33]), separated by intervals of 210 μM, were obtained from each animal. Tissue sections were sequentially treated with 0.3% H_2_O_2_ in PBS for 30 min and 10% normal goat serum in 0.1 M PBS for 30 min at 25 °C and incubated overnight with rabbit anti-glial fibrillary acidic protein (GFAP, 1:1000; Merck Millipore, Temecula, CA, USA), rabbit anti-ionized calcium-binding adapter molecule 1 (Iba-1, 1:500; Wako, Osaka, Japan), rabbit anti-MBP (1:200; Merck Millipore, Temecula, CA, USA), rat anti-Mac3 (1:50; BD Biosciences, San Jose, CA, USA), rabbit anti-Ki67 (1:1000; Abcam, Cambridge, UK), or rabbit anti-doublecortin (DCX, 1:2000; Abcam) at 25 °C. The next day, sections were treated with biotinylated goat anti-rabbit IgG (1:200; Vector, Burlingame, CA, USA) or goat anti-rat IgG (1:200; Vector) for 2 h at 25 °C. Subsequently, sections were treated with streptavidin-peroxidase complex (1:200; Vector) for 2 h at 25 °C. Thereafter, these sections were visualized by reaction with 3,3′-diaminobenzidine tetrahydrochloride (DAB, Sigma) in 0.1 M Tris-HCl buffer (pH 7.2) with or without nickel intensification and mounted on gelatin-coated slides. Sections were dehydrated with serial concentrations of ethanol and mounted using Canada balsam (Kanto Chemical, Tokyo, Japan). For DCX immunohistochemistry, the sections were treated with fluorescein-AffiniPure donkey anti-rabbit IgG (1:200; Jackson ImmunoResearch, West Grove, PA, USA) with 4′,6-diamidino-2-phenylindole (1:1000; Sigma). Thereafter, sections were mounted on gelatin-coated slides using the water-soluble mounting medium, Fluoromount-G^®^ (SouthernBiotech, Birmingham, AL, USA).

### 2.7. Enzyme Immunoassay for Cytokines

Animals (*n* = 5 in each group) were anesthetized with 75 mg/kg alfaxalone (Careside) and 10 mg/kg xylazine (Bayer Korea) at 6 h after ischemia/reperfusion because significant increases in tumor necrosis factor-α (TNF-α) and interleukin (IL)-1β levels were observed in the hippocampus at this time point [34]. The concentrations of inflammatory cytokines, TNF-α and IL-1β, were determined according to the manufacturer’s guidelines and a previous study performed by our colleagues [35]. Briefly, the concentration was measured with optical density at 450 nm and calculated on the basis of linear calibration curves generated with IL-1β and TNF-α standard solutions.

### 2.8. Semi-Quantification of Data

Analyses of GFAP-immunoreactive astrocytes, Iba-1-immunoreactive microglia, MBP-immunoreactive myelin in the hippocampal CA1 region, and DCX-immunoreactive neuroblasts in the hippocampal dentate gyrus, were performed using an image analysis system and ImageJ software (NIH Bethesda, MD, USA) as previously described [25,32]. For objectivity, data were analyzed by two observers for each experiment under blinded conditions. Digital images of the whole dentate gyrus were captured with a BX51 light microscope (Olympus, Tokyo, Japan) equipped with a digital camera (DP72, Olympus) connected to a computer monitor. Images were calibrated into an array of 512 × 512 pixels corresponding to a tissue area of 1200 μM × 900 μM (100× primary magnification). Gray level resolution was 256, and the intensity of GFAP, Iba-1, MBP, and DCX immunoreactivity was evaluated based on the relative optical density (ROD), which was obtained after transformation of the mean gray level using the following formula: ROD = log_10_ (256/mean gray level). The ROD of background staining was determined in unlabeled portions of the sections using Photoshop CC 2018 software (Adobe Systems Inc., San Jose, CA, USA), and this value was subtracted to correct for nonspecific staining using ImageJ software version 1.50. Data were expressed as percentages of the CTL group (set at 100%).

The respective numbers of cresyl violet-stained cells, Ki67-positive nuclei, and Mac3-immunoreactive macrophages were counted using an analysis system with a computer-based, charge-coupled device (CCD) camera (OPTIMAS software version 6.5; Cyber Metrics^®^ Corporation, Phoenix, AZ, USA; magnification, 100×). Counts from all sections were averaged.

### 2.9. Statistical Analysis

The results are shown as the mean ± standard deviation. Statistical analysis of data was performed using one-way analysis of variance (ANOVA). Further comparisons were assessed using Tukey’s multiple-range test in order to elucidate the effects of cuprizone and hypothermia on hippocampal neurogenesis in a transient forebrain ischemia model of gerbils. A *p*-value < 0.05 was considered statistically significant.

## 3. Results

### 3.1. Effects of Cuprizone on Cell Survival after Ischemia in Normothermic and Hypothermic Gerbils

**Figure 2:**
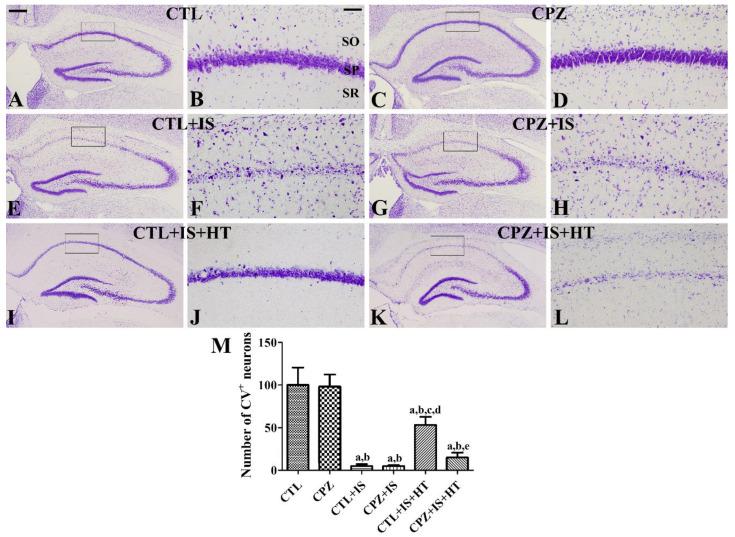
Cresyl violet staining in the hippocampus of the CTL (**A**,**B**), CPZ (**C**,**D**), CTL + IS (**E**,**F**), CPZ + IS (**G**,**H**), CTL + IS + HT (**I**,**J**), and CPZ + IS + HT (**K**,**L**) groups. Cresyl violet-stained neurons were abundantly observed in the hippocampus of the CTL and CPZ groups, while few cresyl violet-stained neurons were detected in the stratum pyramidale (SP) of the CA1 region in the CTL + IS, CPZ + IS, and CPZ + IS + HT groups. Note that there are numerous cresyl violet-stained neurons in the CTL + IS + HT group. SO, stratum oriens; SR, stratum radiatum. Scale bar = 400 μM (**A**,**C**,**E**,**G**,**I**,**K**), 50 μM (**B**,**D**,**F**,**H**,**J**,**L**). (**M**) The number of cresyl violet-stained neurons compared to the CTL group per section, in all the groups, is shown (*n* = 5 per group, ^a^ *p* < 0.05, significantly different from the CTL group; ^b^ *p* < 0.05, significantly different from the CPZ group; ^c^ *p* < 0.05, significantly different from the CTL + IS group; ^d^ *p* < 0.05, significantly different from the CPZ + IS group; ^e^ *p* < 0.05, significantly different from the CTL + IS + HT group). Error bars indicate standard deviation.

In the CTL and CPZ groups, cresyl violet-positive neurons were abundant in the stratum pyramidale of the hippocampal CA1 region (Figure 2A–D). There were no significant differences in the number of cresyl violet-positive cells in the hippocampal CA1 region (Figure 2M). In the CTL + IS group, very few cresyl violet-positive neurons were detected in the stratum pyramidale of the hippocampal CA1 region (Figure 2E,F). In the CPZ + IS group, similar numbers of cresyl violet-positive neurons were observed in the CA1 region compared to the CTL + IS group (Figure 2G,H). The number of cresyl violet-positive cells per section in the CTL + IS and CPZ + IS groups was 5.1% and 4.9%, respectively, of that in the CTL group (Figure 2M). In the CTL + IS + HT group, cresyl violet-positive neurons in the stratum pyramidale were increased compared to those in the CTL + IS group (Figure 2I,J). In this group, the number of cresyl violet-positive neurons was 53.4% of that in the CTL group (Figure 2M). In the CPZ + IS + HT group, the number of cresyl violet-positive neurons in the stratum pyramidale was decreased compared to that in the CTL + IS + HT group (Figure 2K,L), but cresyl violet-positive neurons were abundant compared to those in the CTL + IS group (Figure 2M).

### 3.2. Effects of Cuprizone on Reactive Astrocytosis after Ischemia in Normothermic and Hypothermic Gerbils

**Figure 3:**
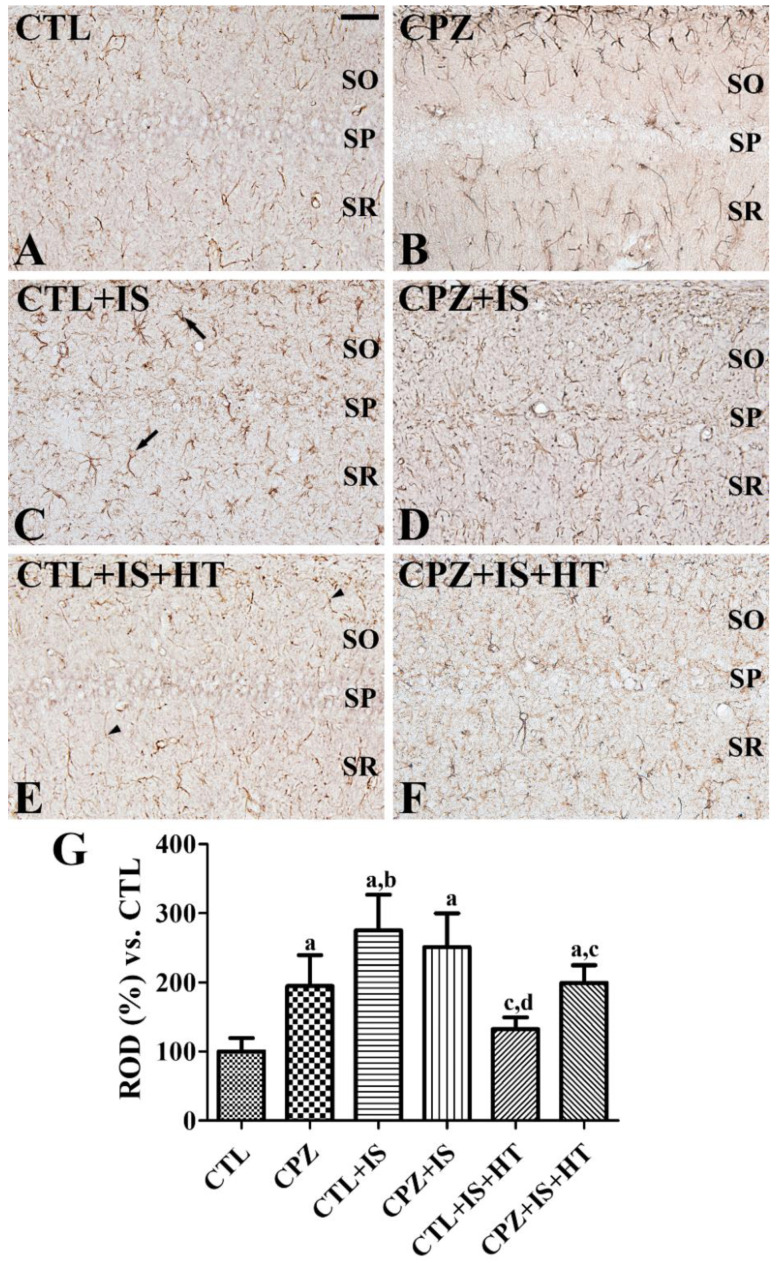
Glial fibrillary acidic protein (GFAP) immunohistochemistry in the gerbil hippocampal CA1 region of the CTL (**A**), CPZ (**B**), CTL + IS (**C**), CPZ + IS (**D**), CTL + IS + HT (**E**), and CPZ + IS + HT (**F**) groups. GFAP-immunoreactive astrocytes were observed in the hippocampal CA1 region. Note that many GFAP-immunoreactive astrocytes have long processes in the CPZ group and punctuated cytoplasm with thickened processes (arrows) in the CTL + IS, CPZ + IS, and CPZ + IS + HT groups, while many astrocytes in the CTL + IS + HT group have thin cytoplasm and processes (arrowheads). SP, stratum pyramidale; SO, stratum oriens; SR, stratum radiatum. Scale bar = 50 μM. (**G**) Relative optical densities (RODs) were expressed as a percentage of the GFAP immunoreactivity detected compared to that of the CTL group in the hippocampal CA1 region for each section (*n* = 5 per group, ^a^ *p* < 0.05, significantly different from the CTL group; ^b^ *p* < 0.05, significantly different from the CPZ group; ^c^ *p* < 0.05, significantly different to the CTL + IS group; ^d^ *p* < 0.05, significantly different from the CPZ + IS group). Error bars indicate standard deviation.

In the CTL group, GFAP-immunoreactive astrocytes of the hippocampal CA1 region had low cytoplasmic levels with long thread-like processes (resting form) (Figure 3A). In the CPZ group, GFAP-positive astrocytes had long processes and GFAP immunoreactivity was significantly increased in the CA1 region compared to that in the control group (Figure 3B,G). In the CTL + IS group, GFAP-positive astrocytes had hypertrophied cytoplasm with thick processes (reactive form) (Figure 3C), and GFAP immunoreactivity was significantly increased in the CA1 region compared to the CTL group (Figure 3G). In the CPZ + IS group, GFAP-positive astrocytes showed similar morphology in the CA1 region to those in the CTL + IS group, and some of them had punctuated cytoplasm (Figure 3D). In this group, GFAP immunoreactivity was similar to that in the CTL + IS group (Figure 3G). In the CTL + IS + HT group, only a few GFAP-positive astrocytes had hypertrophied cytoplasm, but many of them were in the resting form (Figure 3E) and GFAP immunoreactivity was significantly decreased in the CA1 region compared to the CTL + IS group (Figure 3G). In the CPZ + IS + HT group, many GFAP-positive astrocytes in the CA1 region were in the activated form (Figure 3F) and GFAP immunoreactivity was increased compared to the CTL + IS + HT group, although this was not statistically significant (Figure 3G).

### 3.3. Effects of Cuprizone on Reactive Microgliosis after Ischemia in Normothermic and Hypothermic Gerbils

**Figure 4:**
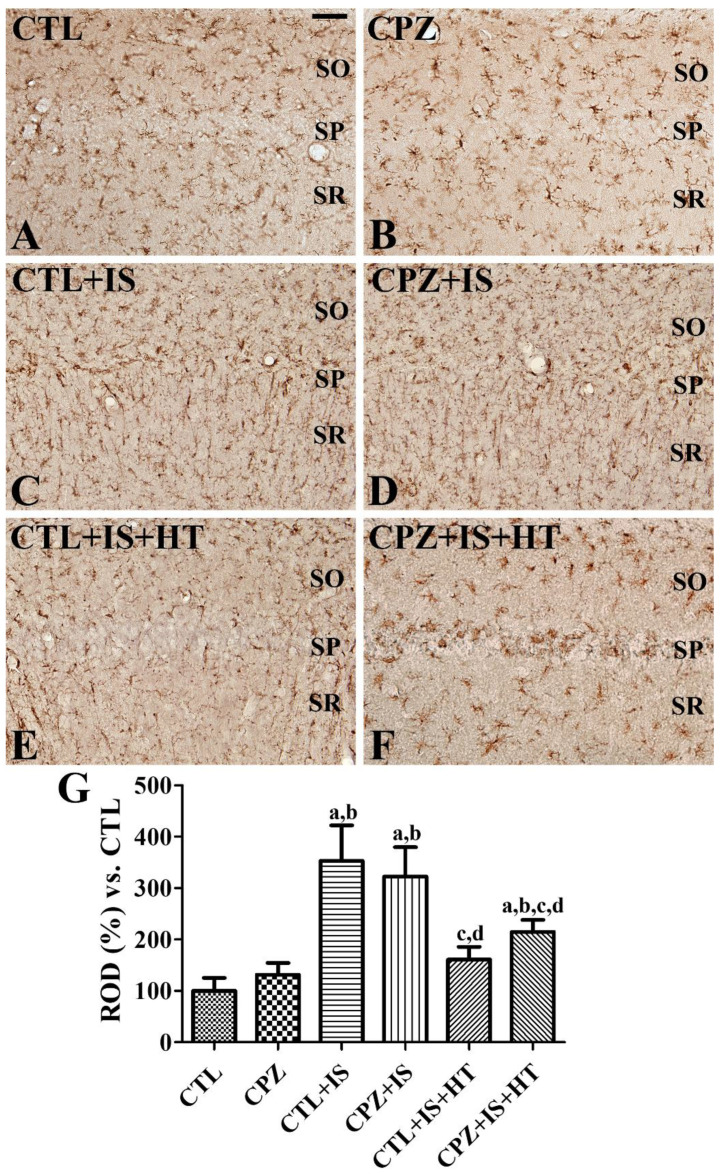
Ionized calcium-binding adapter molecule 1 (Iba-1) immunohistochemistry in the gerbil hippocampal CA1 region of the CTL (**A**), CPZ (**B**), CTL + IS (**C**), CPZ + IS (**D**), CTL + IS + HT (**E**), and CPZ + IS + HT (**F**) groups. Iba-1-immunoreactive microglia were mainly observed in the stratum oriens (SO) and stratum radiatum (SR). Iba-1-immunoreactive microglia have a rounded form cytoplasm in the stratum pyramidale (SP) in the CTL + IS, CPZ + IS, and CPZ + IS + HT groups. Scale bar = 50 μM. (**G**) RODs were expressed as a percentage of the Iba-1 immunoreactivity detected compared to the CTL group in the hippocampal CA1 region for each section (*n* = 5 per group, ^a^ *p* < 0.05, significantly different from the CTL group; ^b^ *p* < 0.05, significantly different from the CPZ group; ^c^ *p* < 0.05, significantly different from the CTL + IS group; ^d^ *p* < 0.05, significantly different from the CPZ + IS group). Error bars indicate standard deviation.

In the CTL group, Iba-1-immunoreactive microglia had low cytoplasmic levels with thin processes (Figure 4A). In the CPZ group, some Iba-1-immunoreactive microglia exhibited hypertrophied cytoplasm and ramified processes (Figure 4B). In this group, Iba-1 immunoreactivity was slightly increased in the hippocampal CA1 region compared to that in the control group (Figure 4G). In the CTL + IS group, Iba-1-immunoreactive microglia exhibited hypertrophied cytoplasm with thickened processes. In addition, round-type Iba-1-immunoreactive microglia were also found in the stratum pyramidale (Figure 4C). In this group, Iba-1 immunoreactivity was significantly increased in the CA1 region compared to the CTL group (Figure 4G). In the CPZ + IS group, the distribution pattern and morphology of Iba-1-immunoreactive microglia were similar to those of the CTL + IS group (Figure 4D), and Iba-1 immunoreactivity was also similarly observed (Figure 4G). In the CTL + IS + HT group, some Iba-1-immunoreactive microglia had hypertrophied cytoplasm, but others had low cytoplasmic levels (Figure 4E) and Iba-1 immunoreactivity was significantly decreased compared to the CTL + IS group (Figure 4G). In the CPZ + IS + HT group, many Iba-1-immunoreactive microglia with hypertrophied cytoplasm were found in the stratum radiatum, while round-type Iba-1-immunoreactive microglia were observed in the stratum pyramidale (Figure 4F). Iba-1 immunoreactivity was slightly higher in the CPZ + IS + HT group than in the CTL + IS + HT group (Figure 4G).

### 3.4. Effects of Cuprizone on Macrophages after Ischemia in Normothermic and Hypothermic Gerbils

**Figure 5:**
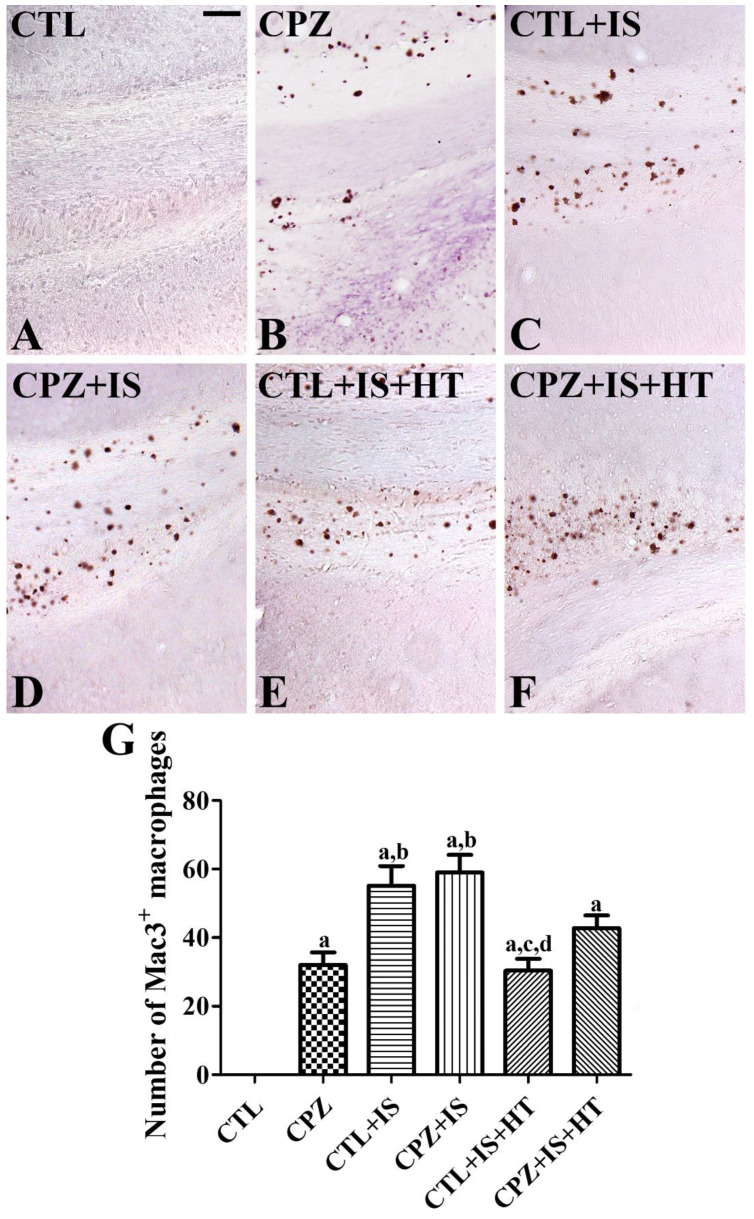
Mac3 immunohistochemistry in the gerbil subcallosal zone of the CTL (**A**), CPZ (**B**), CTL + IS (**C**), CPZ + IS (**D**), CTL + IS + HT (**E**), and CPZ + IS + HT (**F**) groups. Mac3-immunoreactive macrophages were mainly observed in the subcallosal zone after CPZ treatment and/or ischemic damage. Mac3-immunoreactive macrophages were most abundant in the CPZ + IS group and lowest in the CPZ or CTL + IS + HT group. Scale bar = 50 μM. (**G**) The number of Mac3-immunoreactive macrophages per section in each group is shown (*n* = 5 per group, ^a^ *p* < 0.05, significantly different from the CTL group; ^b^ *p* < 0.05, significantly different from the CPZ group; ^c^ *p* < 0.05, significantly different from the CTL + IS group; ^d^ *p* < 0.05, significantly different from the CPZ + IS group). Error bars indicate standard deviation.

**Figure 6:**
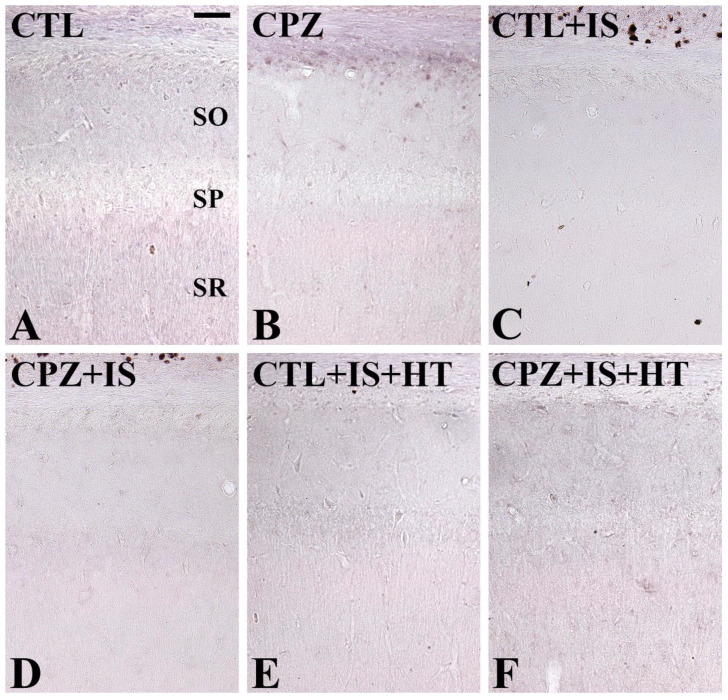
Mac3 immunohistochemistry in the gerbil hippocampal CA1 region of the CTL (**A**), CPZ (**B**), CTL + IS (**C**), CPZ + IS (**D**), CTL + IS + HT (**E**), and CPZ + IS + HT (**F**) groups. Mac3-immunoreactive macrophages were not detectable in the hippocampal CA1 region of all groups.

In the CTL group, Mac3-immunoreactive macrophages were not detectable in the hippocampal CA1 region and subcallosal zone of the cortex (Figure 5A and Figure 6A). In the CPZ group, Mac3-immunoreactive macrophages were found in the subcallosal zone (Figure 5B), but not in CA1 (Figure 6B), and the number of Mac3-immunoreactive macrophages was 32.0 per section (Figure 5G). In the CTL + IS group, Mac3-immunoreactive macrophages were abundantly found in the subcallosal zone and (Figure 5C), but not in CA1 (Figure 6C), with a pattern similar to that in the CPZ-treated group. In this group, the number of Mac3-immunoreactive macrophages per section was 55.2, which was significantly higher than that in the CPZ group (Figure 5G). In the CPZ + IS group, the number of Mac3-immunoreactive macrophages was slightly higher in the subcallosal zone than in the CTL + IS group (Figure 5D,G), while in the CA1 region, Mac3-immunoreactive macrophages were not detected (Figure 6D). In the CTL + IS + HT group, Mac3-immunoreactive macrophages were significantly decreased compared to the CTL + IS or CPZ + IS groups, and the number per section was 30.4 (Figure 5E,G). In the CPZ + IS + HT group, Mac3-immunoreactive cells showed higher numbers compared to those in the CTL + IS + HT or CPZ + IS groups in the subcallosal zone, and the number of Mac3-immunoreactive cells per section was 42.8 (Figure 5F,G). However, Mac3-immunoreactive structures were not detected in CA1 of the CTL + IS + HT and CPZ + IS + HT groups (Figure 6E,F).

### 3.5. Effects of Cuprizone on MBP Expression after Ischemia in Normothermic and Hypothermic Gerbils

**Figure 7:**
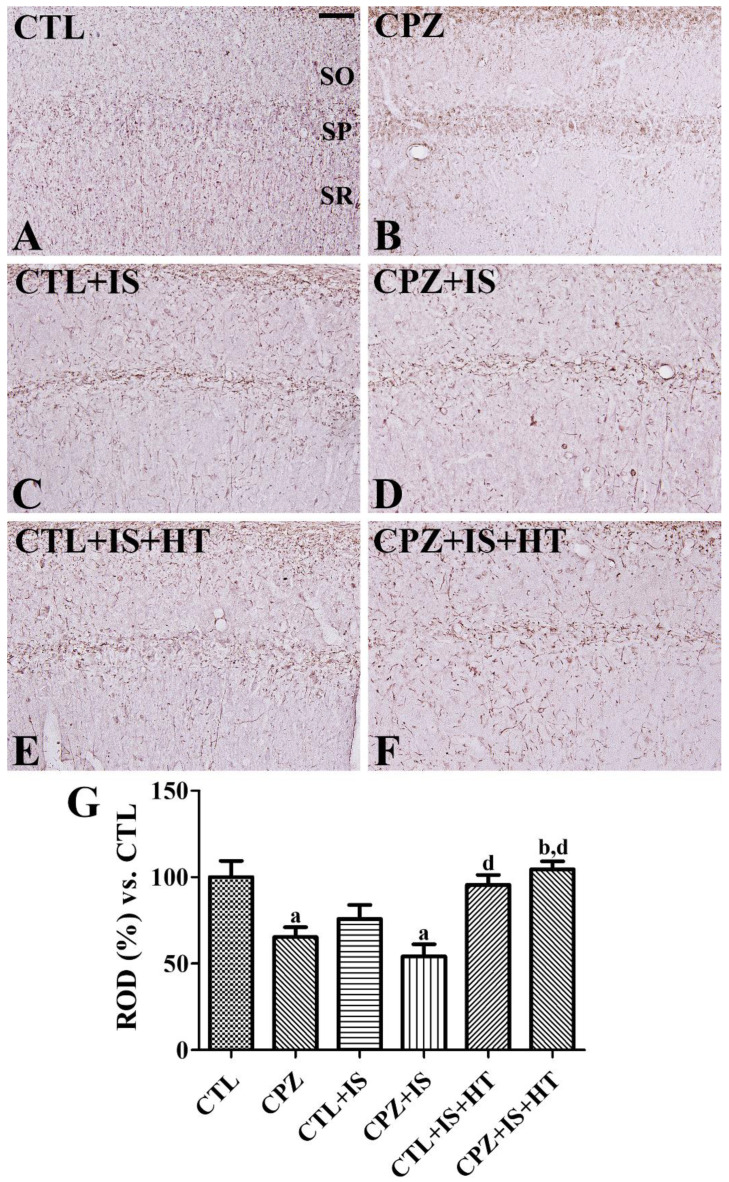
Myelin basic protein (MBP) immunohistochemistry in the gerbil hippocampal CA1 region of the CTL (**A**), CPZ (**B**), CTL + IS (**C**), CPZ + IS (**D**), CTL + IS + HT (**E**), and CPZ + IS + HT (**F**) groups. MBP-immunoreactive structures were found in the stratum oriens (SO), radiatum (SR), and pyramidale (SP). Note that MBP-immunoreactive structures are most prominent in the CTL group and exhibit lowest levels in the CPZ + IS group. Scale bar = 50 μM. (**G**) RODs are expressed as a percentage of the MBP immunoreactivity detected compared to the CTL group in the hippocampal CA1 region for each section (*n* = 5 per group, ^a^ *p* < 0.05, significantly different from the CTL group; ^b^ *p* < 0.05, significantly different from the CPZ group; ^d^ *p* < 0.05, significantly different from the CPZ + IS group). Error bars indicate standard deviation.

In the CTL group, MBP-immunoreactive structures were found in the hippocampal CA1 region (Figure 7A). In the CPZ group, MBP immunoreactivity was significantly decreased to 65.4% of the CTL group (Figure 7B,G). In the CTL + IS group, MBP-immunoreactive structures decreased to 75.8% of the CTL group in the CA1 region (Figure 7C,G). In the CPZ + IS group, fewer MBP-immunoreactive structures were found in the CA1 region and MBP immunoreactivity was significantly decreased compared to that in the CTL group and was 54.2% of the CTL group (Figure 7D,G). In the CTL + IS + HT and CPZ + IS + HT groups, MBP immunoreactivity was higher than those in the CTL + IS and CPZ + IS groups (Figure 7E,F). Notably, MBP immunoreactivity in the CTL + IS + HT and CPZ + IS + HT groups was significantly increased in CA1 compared to that in the CPZ + IS group (Figure 7G). However, MBP immunoreactivity did not show any significant changes in CA1 between the CTL + IS + HT and CPZ + IS + HT groups (Figure 7G).

### 3.6. Effects of Cuprizone on IL-1β and TNF-α after Ischemia in Normothermic and Hypothermic Gerbils

**Figure 8:**
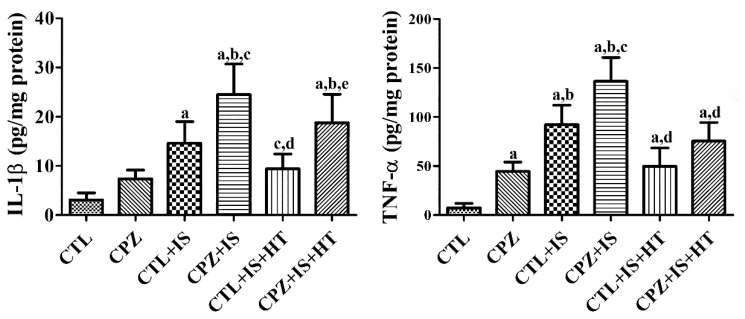
IL-1β and TNF-α levels in the gerbil hippocampal homogenates of the CTL, CPZ, CTL + IS, CPZ + IS, CTL + IS + HT, and CPZ + IS + HT groups 6 h after ischemia/reperfusion. Note that IL-1β and TNF-α levels were highest in the CPZ + IS group and lowest in the CTL + IS + HT group (*n* = 5 per group, ^a^ *p* < 0.05, significantly different from the CTL group; ^b^ *p* < 0.05, significantly different from the CPZ group; ^c^ *p* < 0.05, significantly different from the CTL + IS group; ^d^ *p* < 0.05, significantly different from the CPZ + IS group; ^e^ *p* < 0.05, significantly different from the CTL + IS + HT group). Error bars indicate standard deviation.

In the CPZ group, IL-1β and TNF-α levels were increased in the hippocampal homogenates compared to those in the CTL group. In particular, TNF-α levels were significantly elevated by 594.4% of the CTL group. In the CTL + IS group, IL-1β and TNF-α levels were significantly increased to 476.2% and 1232.7% of those of the CTL group in the hippocampal homogenates, respectively. In the CPZ + IS group, IL-1β and TNF-α levels were further increased in the hippocampal homogenates and showed statistical significance compared to those in the CTL or CTL + IS groups, respectively. However, the CTL + IS + HT group showed a significant reduction in IL-1β and TNF-α levels to 64.3% and 53.9% of those of the CTL + IS group, respectively. In the CPZ + IS + HT group, IL-1β and TNF-α levels increased compared to those in the CTL + IS + HT group, but the levels were respectively lower than those in the CPZ + IS group (Figure 8).

### 3.7. Effects of Cuprizone on Proliferating Cells after Ischemia in Normothermic and Hypothermic Gerbils

**Figure 9:**
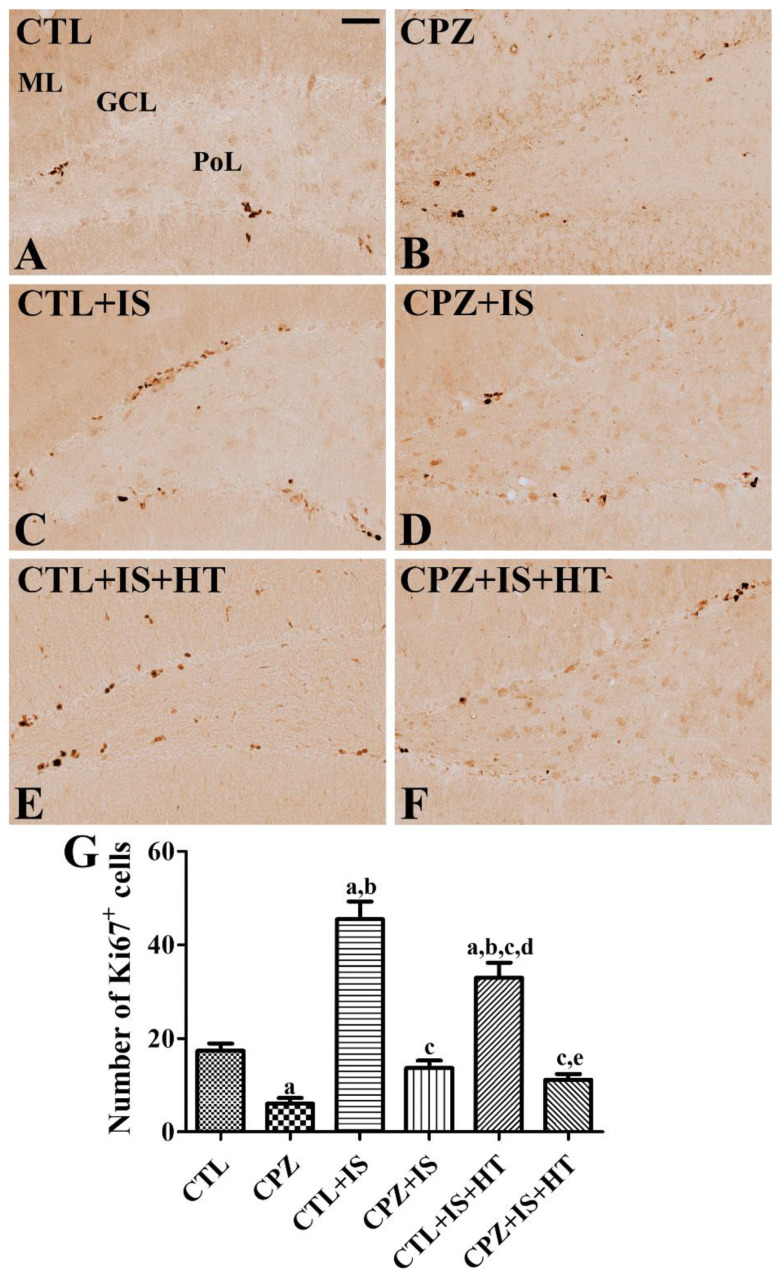
Ki67 immunohistochemistry in the gerbil hippocampal dentate gyrus of the CTL (**A**), CPZ (**B**), CTL + IS (**C**), CPZ + IS (**D**), CTL + IS + HT (**E**), and CPZ + IS + HT (**F**) groups. Ki67-positive nuclei were observed in the subgranular zone of the dentate gyrus. Ki67-positive nuclei were most abundant in the CTL + IS group and fewest in the CPZ group. GCL, granule cell layer; ML, molecular layer; PoL, polymorphic layer. Scale bar = 50 μM. (**G**) The number of Ki67-positive nuclei per section in each group is shown (*n* = 5 per group, ^a^ *p* < 0.05, significantly different from the CTL group; ^b^ *p* < 0.05, significantly different from the CPZ group; ^c^ *p* < 0.05, significantly different from the CTL + IS group; ^d^ *p* < 0.05, significantly different from the CPZ + IS group; ^e^ *p* < 0.05, significantly different from the CTL + IS + HT group). Error bars indicate standard deviation.

In the CTL group, Ki67-positive cells were detected in the dentate gyrus (Figure 9A), and the number of Ki67-positive cells per section was 17.4 (Figure 9G). In the CPZ group, fewer Ki67-positive cells were found in the subgranular zone of the dentate gyrus, averaging 6.0 per section (Figure 9B,G). In the CTL + IS group, the number of Ki67-positive cells was increased compared to that in the CTL group, and the number per section was 45.5 (Figure 9C,G). In the CPZ + IS group, the number of Ki67-positive cells was significantly decreased in the dentate gyrus compared to the CTL + IS group (Figure 9D), and a similar number of Ki67-positive cells was found compared to the CTL group (Figure 9G). In the CTL + IS + HT group, the number of Ki67-positive cells was significantly reduced compared to the CTL + IS group, and the number per section was 33.0 (Figure 9E,G). In the CPZ + IS + HT group, a few Ki67-positive cells, averaging 11.1 per section, were found in the dentate gyrus (Figure 9F,G).

### 3.8. Effects of Cuprizone on Differentiated Neuroblasts after Ischemia in Normothermic and Hypothermic Gerbils

**Figure 10:**
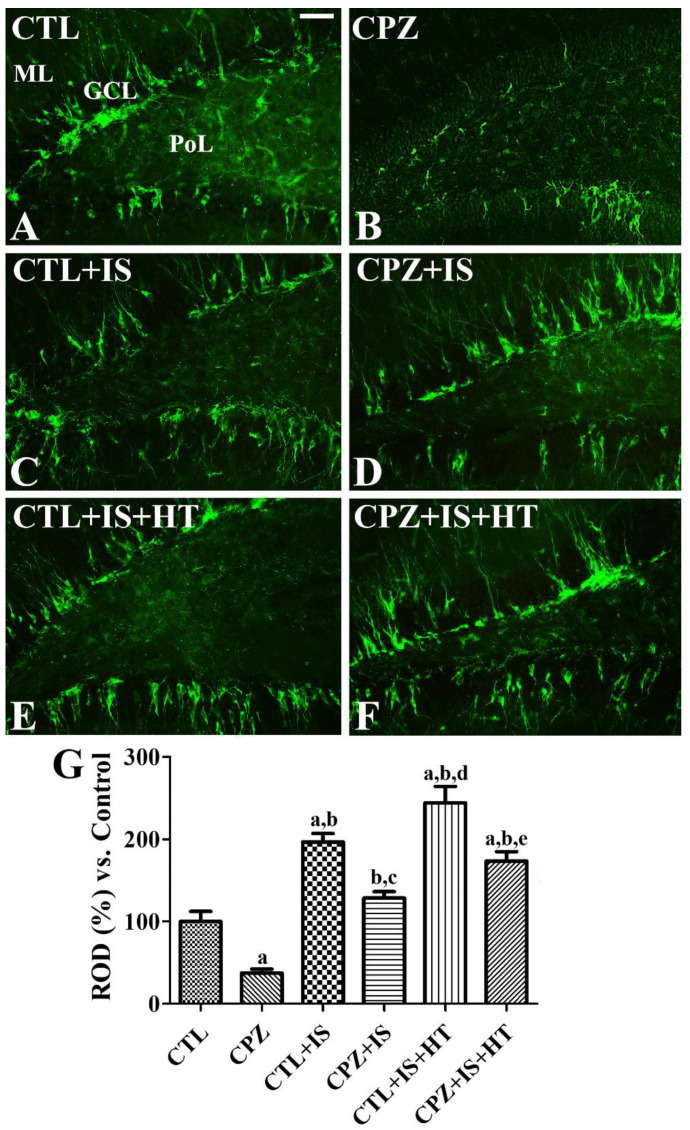
Doublecortin (DCX) immunohistochemistry in the gerbil hippocampal dentate gyrus of the CTL (**A**), CPZ (**B**), CTL + IS (**C**), CPZ + IS (**D**), CTL + IS + HT (**E**), and CPZ + IS + HT (**F**) groups. DCX-immunoreactive neuroblasts have a round cytoplasm with dendrites that extend into the molecular layer (ML). Note that DCX-immunoreactive structures were most abundant in the CTL + IS + HT group and fewest in the CPZ group. GCL, granule cell layer; PoL, polymorphic layer. Scale bar = 50 μM. (**G**) RODs were expressed as a percentage of the DCX immunoreactivity detected compared with that of the CTL group in the hippocampal dentate gyrus for each section (*n* = 5 per group, ^a^ *p* < 0.05, significantly different from the CTL group; ^b^ *p* < 0.05, significantly different from the CPZ group; ^c^ *p* < 0.05, significantly different from the CTL + IS group; ^d^ *p* < 0.05, significantly different from the CPZ + IS group; ^e^ *p* < 0.05, significantly different from the CTL + IS + HT group). Error bars indicate standard deviation.

In the CTL group, DCX-immunoreactive neuroblasts were detected in the dentate gyrus (Figure 10A). In the CPZ group, a few DCX-immunoreactive neuroblasts were found in the dentate gyrus and DCX immunoreactivity was significantly decreased to 37.4% of the CTL group (Figure 10B,G). In the CTL + IS group, DCX-immunoreactive neuroblasts were abundantly found in the dentate gyrus, and the mean percentage of ROD in this group was 196.9% of that in the CTL group (Figure 10C,G). DCX immunoreactivity in the CPZ + IS group was similar to that observed in the CTL group (Figure 10D). In the CTL + IS + HT group, DCX immunoreactivity was the highest among the groups (Figure 10E,G), while in the CPZ + IS + HT group, DCX-immunoreactive neuroblasts were less abundant than in the CTL + IS + HT group (Figure 10F). The mean percentages of ROD in the CTL + IS + HT and CPZ + IS + HT groups were 244.4% and 173.7%, respectively, compared with 100% in the CTL group (Figure 10G).

## 4. Discussion

Interruptions in the blood supply to the brain, as can occur during cardiac arrest, can lead to neuronal death and subsequent loss of brain function [2,3,4]. Cuprizone causes demyelination in the hippocampus and a reduction in the number of neural progenitor cells in the subgranular zone of the dentate gyrus [25,36]. In the present study, we investigated the effects of cuprizone on neuronal degeneration in ischemic damage based on cresyl violet staining and immunohistochemistry for GFAP and Iba-1 in the hippocampal CA1 region. Cuprizone treatment of normal gerbils did not show any significant changes in cresyl-violet positive cells in the hippocampus. Transient forebrain ischemia in gerbils selectively induced neuronal damage in the CA1 region, but not in the CA2-3 regions, on the fourth day after ischemic surgery. Transient forebrain ischemia showed massive neuronal damage in the hippocampal CA1 region of the CTL + IS and CPZ + IS groups, and there were no differences in the number of cresyl violet-positive cells between groups. However, Wang et al. previously demonstrated that cuprizone treatment exacerbates brain damage caused by middle cerebral artery occlusion in mice [30]. The discrepancies may be associated with cell death patterns in the study. Transient ischemic models in gerbils cause selective neuronal damage (about 95%) in the hippocampal CA1 region, while non-selective and broad neuronal damage occurs in focal ischemia by middle cerebral artery occlusion. Hypothermia prevents neurons from ischemic damage in the hippocampal CA1 region, consistent with the results of previous studies [15,17,18]. In the present study, cuprizone treatment decreased hypothermia-induced neuroprotection against ischemic damage. This result suggests that cuprizone-induced demyelination affects the neuroprotective actions of hypothermia in ischemic gerbils.

Reactive astrocytes are one of the main sources of CCL_2_, a pro-inflammatory cytokine that plays a crucial role in the recruitment and activation of macrophages [37]. In addition, astrocytes are one of the primary components of the blood–brain barrier and the activation of astrocytes may disrupt the barrier, resulting in the entry of toxicants into the brain, in experimental autoimmune encephalomyelitis [38]. In this study, we evaluated the expression of GFAP-immunoreactive astrocytes and Iba-1-immunoreactive microglia in the hippocampal CA1 region to confirm cuprizone’s effect on brain ischemic injury. Cuprizone treatment significantly increased the number of GFAP immunoreactive astrocytes, but not of Iba-1-immunoreactive microglia, in the hippocampal CA1 region, although Iba-1 immunoreactivity was increased in the CPZ group. This result is consistent with previous studies showing that GFAP-immunoreactive astrocytes are abundantly expressed in the septal nucleus, corpus callosum, and hippocampus [39,40,41]. In addition, Iba-1 immunoreactivity was prominently increased in the hippocampus [40,41]. Transient forebrain ischemia showed hypertrophied cytoplasm in astrocytes and microglia in the hippocampal CA1 region of the CTL + IS and CPZ + IS groups, and there were no remarkable morphological differences in GFAP and Iba-1 immunoreactivity in the CA1 region between the CTL + IS and CPZ + IS groups. Cuprizone by itself, however, has been shown to increase the activation of astrocytes and microglia in the hippocampus [26,42]. Hypothermia treatment significantly reduced the ischemia-induced reactive astrocytosis and microgliosis in the hippocampal CA1 region, but GFAP and Iba-1 immunoreactivity was significantly increased in the hippocampal CA1 region compared to the CTL group. This result is consistent with a previous study demonstrating that therapeutic hypothermia significantly decreased the intensity of GFAP and Iba-1 staining in hippocampal CA2/CA3 regions in correlation with a reduced injury score [43]. Mild hypothermia in global cerebral ischemia has been shown to decrease microglial activation and translocation of nuclear factor-kappa B [44]. However, Kumar and Evans demonstrated that hypothermic ischemia in gerbils showed no activation of microglia seven days after ischemia/reperfusion, based on isolectin B4 histochemical staining [45]. In the present study, demyelination induced by cuprizone treatment significantly increased GFAP and Iba-1 immunoreactivity in the hippocampal CA1 region, pointing to the promotion of inflammatory responses in ischemic gerbils exposed to cuprizone. In addition, Iba-1-immunoreactive microglia in the CPZ + IS + HT group had a round form of cytoplasm and were concentrated in the stratum pyramidale. This may be due to the engulfment of dying pyramidal neurons in the hippocampal CA1 region [46]. However, we could not observe the round-form microglia in the CTL + IS + HT group. These results suggest that cuprizone diminishes hypothermia-induced neuroprotection against ischemic damage in the gerbil hippocampal CA1 region. Chronic treatment with methylprednisolone exacerbates cuprizone-induced demyelination with elevated levels of inflammatory markers [47]. In addition, the overexpression of C3a and C5a significantly increased demyelination severity in the corpus callosum after cuprizone treatment [48].

Mac3, also known as CD107b and lysosome-associated membrane protein 2, contributes to immune functions by presenting processed antigens and activating T cells [49,50]. Mac3 is also expressed on the surface of mouse mononuclear phagocytes. In the present study, we observed Mac3-immunoreactive macrophages in the corpus callosum and subcallosal zone in the CPZ group, but we did not observe any expression of Mac3-immunoreactive macrophages in the hippocampal CA1 region. This result is consistent with a previous study showing that Mac-immunoreactive macrophages are found in the corpus callosum that has been exposed to cuprizone treatment for 6 weeks in C57BL/6 mice [51]. We also observed significant increases in Mac3-immunoreactive macrophages in the subcallosal zone, but not in the hippocampus, two weeks after ischemia/reperfusion. This result is consistent with previous studies showing that Mac3 expression is significantly increased in the cerebral cortex after traumatic brain injury [52] and 24 h after permanent middle cerebral artery occlusion [53]. In the present study, CTL + IS and CPZ + IS groups showed the highest levels of Mac3-immunoreactive macrophages and the lowest levels in the subcallosal zone of the CTL + IS + HT group. This result suggests that hypothermia significantly decreases macrophage infiltration in the subcallosal zone.

In the present study, we also observed MBP immunoreactivity in the hippocampal CA1 region because MBP is one of the main components in the myelin sheath. The CPZ group showed a significant reduction in MBP immunoreactive structures in the hippocampal CA1 region, suggesting that gerbils are susceptible to cuprizone-induced myelination. The CTL + IS group showed lower levels of MBP immunoreactivity in the hippocampal CA1 region compared to that in the CTL group. This result is consistent with previous studies that showed that the MBP level is decreased two weeks after ischemia in gerbils [54]. In addition, an in vitro study also demonstrated that oligodendrocytes reversibly decrease the production of MBP after hypoxic conditions [55]. In the present study, cuprizone treatment further decreased MBP immunoreactivity in the hippocampus and showed significant reduction between the CTL and CPZ + IS groups, and hypothermia attenuated the reduction in MBP immunoreactivity after ischemia/reperfusion. This result suggests that cuprizone facilitates the demyelination induced by ischemia, and that hypothermia attenuates the demyelination induced by ischemia and/or cuprizone. Furthermore, MBP staining also suggests that hypothermia increases white matter integrity in mice after inducing the hypoxia-ischemia model [43] and after middle cerebral artery occlusion [56].

In the present study, the CPZ group showed elevated IL-1β and TNF-α levels in the hippocampal homogenates, and several studies have demonstrated increases in TNF-α levels [57,58] and IL-1β levels in brain homogenates [39]. Inflammation induced by ischemia upregulates cytokines in the hippocampus, including in gerbils [34]. The most important cytokines related to ischemia-induced inflammation are IL-1, TNF-α, IL-6, IL-10, and transforming growth factor-β [59]. Inhibition of the IL-1 receptor or TNF-α significantly reduces brain damage induced by ischemia, while the overexpression or treatment of IL-1 or TNF-α worsens ischemic damage [60,61,62]. In the present study, we observed IL-1β and TNF-α levels in hippocampal homogenates 6 h after ischemia/reperfusion. Transient forebrain ischemia significantly increased IL-1β and TNF-α levels in hippocampal homogenates, and cuprizone treatment aggravated the increase in IL-1β and TNF-α in the hippocampus after ischemia. However, hypothermia significantly reduced IL-1β and TNF-α levels in the hippocampal homogenates of ischemic insult and/or cuprizone treatment. This result is consistent with a previous study showing that hypothermia could decrease detrimental M1 phenotype microglia and promote healthy M2 phenotype microglia [56]. Collectively, these results suggest that demyelination attenuates the neuroprotective and anti-inflammatory effects of hypothermia therapy against ischemic damage.

Recent studies have demonstrated that brain ischemic injuries dramatically increase neurogenesis in the subgranular zone of the dentate gyrus in rodents [63,64,65]. In the present study, we performed immunohistochemical staining for Ki67 and DCX to confirm the effect of cuprizone on ischemia-induced hippocampal neurogenesis. The CPZ group showed a significant reduction in cell proliferation and neuroblast differentiation in the dentate gyrus compared to that in the CTL group. This result is in agreement with previous studies showing that neurogenesis is decreased in cuprizone-treated mice [25,66,67]. We also observed that transient forebrain ischemia significantly increased cell proliferation and neuroblast differentiation in the dentate gyrus of the hippocampus, consistent with previous studies [68,69,70,71]. Cuprizone exposure to dams significantly decreased progenitor cells in the dentate gyrus of their offspring [72], and oral administration of cuprizone for four weeks also decreased progenitor cells in the dentate gyrus of rats [36] and mice [25]. In the present study, we observed that cuprizone treatment significantly reduced the increase in proliferating cells and differentiated neuroblasts after ischemia in the dentate gyrus, resulting in similar levels to that in the control group. A recent study demonstrated that focal ischemia shows limited neurogenesis at 6 weeks after ischemia in the subventricular zone of the lateral ventricle after cuprizone treatment [67]. In contrast, we sacrificed the animals 2 weeks after ischemia because cell proliferation and neuroblast differentiation in gerbils is significantly increased in the dentate gyrus [68]. Hypothermia treatment significantly decreased the number of proliferating cells in the dentate gyrus after ischemia/reperfusion, while differentiated neuroblasts were significantly increased in the hypothermia-treated ischemic group. This result suggests that hypothermia treatment facilitates the differentiation of progenitor cells, but proliferation is inhibited two weeks after ischemia. There have been conflicting reports of the effects of hypothermia on hippocampal neurogenesis on the dentate gyrus. Hypothermia significantly increased the number of newly generated granule cells in the dentate gyrus four weeks after ischemia [21] and enhanced differentiation of precursor cells and angiogenesis [19]. However, Lasarzik et al. did not find any direct evidence for the increase in differentiated neuroblasts and newly generated granule cells in the dentate gyrus [73]. In addition, Sandu et al. observed an increase in the formation of blood vessels in the penumbra region, but neurogenesis was not enhanced in aged rats [23]. Cuprizone-induced demyelination also decreased the number of differentiated neuroblasts induced by ischemia and hypothermia treatment. In addition, proliferating cells were dramatically decreased in the dentate gyrus. These results suggest that cuprizone-induced demyelination affects the regeneration process induced by hypothermia therapy after ischemia.

In conclusion, cuprizone-induced demyelination does not cause neuronal damage in the hippocampus of normal animals and does not facilitate neuronal damage in the hippocampus after transient forebrain ischemia, but alters the therapeutic potential of hypothermia in the ischemic hippocampus. In addition, cuprizone-induced demyelination inhibits cell proliferation and neuroblast differentiation in the dentate gyrus after ischemia and hypothermic therapy.
